# Targeting Cognitive Dysfunction in Spinocerebellar Ataxia Type 2 Through Digital Cognitive Training

**DOI:** 10.1007/s12311-025-01956-2

**Published:** 2026-03-02

**Authors:** Andressa Aline Vieira, Renata Barreto Tenório, Salmo Raskin, Karla Patricia Figueroa, Stefan M Pulst, Amer Cavalheiro Handam, Hélio Afonso Ghizoni Teive, Carlos Henrique Ferreira Camargo

**Affiliations:** 1 Neurological Diseases Group, Postgraduate Program in Internal Medicine, Internal Medicine Department, Hospital de Clínicas, Federal University of Paraná, Curitiba, Paraná Brazil; 2 Neurogenetic Service, Instituto de Neurologia de Curitiba, Curitiba, Paraná Brazil; 3 Postgraduate Program in Child and Adolescent, Department of Pediatrics, Federal University of Paraná, Curitiba, Paraná Brazil; 4 Department of Neurology, University of Utah, Salt Lake City, UT USA; 5 Department of Psychology, Federal University of Paraná, Curitiba, Paraná Brazil; 6 Movement Disorders Sector, Neurology Service, Internal Medicine Department, Hospital de Clínicas, Federal University of Paraná, Curitiba, Paraná Brazil

**Keywords:** Spinocerebellar ataxias, Cognitive training, Mental status and dementia tests, Neurobehavioral manifestations, Cerebellar cognitive affective syndrome, Cognitive dysfunction

## Abstract

Background: Spinocerebellar ataxia type 2 (SCA2) is a neurodegenerative disorder characterized not only by motor impairment but also by significant cognitive decline. While cognitive training programs have shown promising results in other neurological conditions, evidence regarding their efficacy in SCA2 remains scarce. Objective: To assess cognitive performance in patients with SCA2 and to evaluate the impact of a digital cognitive training program on cognitive functions. Methods: Twelve SCA2 patients (50% male; 46 ± 11.51 years old; 8 ± 3.69 years of education) underwent a comprehensive neuropsychological assessment before and after a six-month cognitive training program delivered through the NeuroNation^®^ digital platform. The evaluated cognitive functions included memory, executive function, language, complex attention, and perceptual–motor abilities. A control group of 12 healthy individuals from the community was selected and matched for sex, age, and education (50% male; 47 ± 11.68 years old; 8 ± 3.85 years of education). Results: SCA2 patients exhibited significantly lower performance across multiple cognitive functions compared to controls, with marked impairments in verbal and visual memory, verbal fluency, attention, and executive function. Following the intervention, significant improvements were observed in several memory subcomponents, including incidental, immediate, delayed, and recognition memory. However, changes in executive functions were limited or absent. No strong correlations were found between game difficulty level and neuropsychological test outcomes. Conclusion: SCA2 patients demonstrated significant cognitive impairments, particularly in memory-related functions, and responded to digital cognitive training. These findings suggest the potential role of targeted interventions in supporting cognitive function in SCA2.

**Supplementary Information:**

The online version contains supplementary material available at 10.1007/s12311-025-01956-2.

## Introduction

Spinocerebellar ataxias (SCAs) represent a heterogeneous group of neurodegenerative disorders that primarily affect the cerebellum and its neural pathways. Spinocerebellar Ataxia Type 2 (SCA2) is characterized by a CAG trinucleotide repeat expansion in the ATXN2 gene. This genetic alteration results in progressive degeneration of the cerebellum, basal ganglia, and additional central nervous system structures [1, 2]. In addition to motor manifestations such as ataxia, dysarthria, and oculomotor disturbances, recent investigations have identified significant cognitive deficits in patients with SCA2, particularly involving executive function, memory, and verbal fluency [3–6].

For decades, the cerebellum was regarded primarily as a structure subserving motor coordination and postural control. However, advances in clinical neuroscience have elucidated its essential involvement in higher-order cognitive, affective, and behavioral functions [4, 7–9]. Schmahmann and Sherman [10, 11] were seminal in delineating the Cerebellar Cognitive Affective Syndrome (CCAS), characterized by deficits in executive function, language, visuospatial abilities, and affective or personality disturbances following cerebellar injury [10, 11]. Functional neuroimaging and clinical investigations have corroborated this framework, mapping discrete cerebellar regions interconnected with prefrontal, parietal, and limbic circuits [7–9, 12]. Within the context of SCA2, several pathophysiological mechanisms have been posited to underlie cognitive dysfunction, including disruption of cortico-ponto-cerebello-thalamo-cortical pathways [10], fronto-subcortical loops [13], degeneration of cholinergic projections [14], and crossed cerebellar diaschisis [15]. Collectively, these findings indicate that cognitive decline in SCA2 arises from multifactorial, distributed network dysfunction selectively affecting specific cognitive functions [16].

Digital cognitive training, also referred to as “brain games,” aims to enhance specific cognitive abilities or overall cognitive performance by repeatedly engaging users in targeted tasks over time [17]. These platforms simulate real-life activities requiring coordination, precision, planning, organization, and repetition, allowing users to practice daily tasks and gain a sense of accomplishment virtually. Depending on the exercise design, these platforms target cognitive functions such as processing speed, working memory, inhibitory control, cognitive flexibility, visuospatial skills, problem-solving, and sustained or divided attention. Many platforms also provide tasks to strengthen executive functions—such as planning, monitoring, error correction, and strategic reasoning—while others focus on verbal and nonverbal memory systems through recall, recognition, and sequencing tasks. This positive reinforcement may enhance real-world performance [17]. Based on neuroplasticity, these platforms aim to stimulate cognitive functions whose improvement could support daily functioning and overall quality of life [17–19]. Although promising outcomes have been reported in individuals with mild cognitive impairment and Parkinson’s disease (PD) [20–23], a noticeable gap persists in terms of applicability for individuals with ataxia, particularly SCA2.

Accordingly, in light of the identified gap in prior research and the potential efficacy of cognitive training observed in other neurological disorders, this study sought to systematically evaluate cognitive impairments in patients with SCA2 and to subsequently assess their response to a targeted cognitive training intervention administered via a digital platform.

## Methods

### Patient Selection

Patients were recruited from the Spinocerebellar Ataxia database at the Ataxia Outpatient Clinic, located at the Complexo Hospital de Clínicas, Federal University of Paraná (CHC-UFPR). Among the registered SCA cases, 30 individuals with genetically confirmed SCA2, defined as having 35 or more CAG repeats in at least one ATXN2 allele, were contacted between August 2020 and August 2022.

Inclusion criteria were: (a) age > 18 years; (b) completion of genetic testing, neuroimaging, and laboratory evaluations to exclude secondary causes of ataxia; and (c) regular follow-up at the Ataxia Clinic with the ability to participate in assessments and training. Exclusion criteria included other neurological or medical conditions affecting speech, cognition, or behavior, or a score below 10 on the Montreal Cognitive Assessment – Basic (MoCA-B) [24].

After excluding those who met exclusion criteria, those who had died (including during the COVID-19 pandemic), and those who declined participation, the final sample consisted of 12 patients with SCA2. A control group of 12 healthy individuals, matched for age, education, and clinical comorbidities, was selected from the community. The same exclusion criteria applied to the patient group were used for the controls.

### Ethical Considerations

This study was approved by the Research Ethics Committee (CEP) of CHC-UFPR (CAAE: 06128812.0.0000.0096). All participants provided informed consent prior to inclusion.

### Clinical and Neuropsychological Assessment

A structured interview was conducted to collect demographic data, including sex, age at the first interview, educational level, and occupation. Detailed medical history included age, disease duration, symptom characteristics, comorbidities, and current medications. Genetic test results, neuroimaging findings, and other complementary data were extracted from medical records.

Neurological examination included:

- Assessment and Rating of Ataxia (SARA): The total SARA score ranges from 0 (no ataxia) to 40 (most severe ataxia), divided into eight items: (1) gait (0–8); (2) stance (0–6); (3) sitting balance (0–4); (4) speech disturbance (0–6); (5) finger chase (0–4); (6) nose-finger test (0–4); (7) fast alternating hand movements (0–4); (8) heel-shin slide (0–4). Kinetic limb functions (items 5–8) are scored independently for each side, and the mean score from both sides contributes to the total [25].

Neuropsychological assessments were conducted pre- and post-intervention using neurocognitive tests and scales.

Neurocognitive tests:

- Montreal Cognitive Assessment – Basic (MoCA-B): Screening measure sensitive to mild cognitive impairment, assessing executive functions, attention, memory, language, abstraction, visuospatial abilities, and orientation. Total score = 30; scores ≤ 24 indicate clinically relevant impairment [24].
- Verbal Fluency Test (semantic and phonemic): Participants generate words within one minute by semantic category (animals) and by initial letter (F, A, S). Assesses lexical retrieval, semantic memory, inhibitory control, working memory, and cognitive flexibility by generating words within semantic and phonemic constraints [26].
- Trail Making Test (TMT), Parts A and B: Part A evaluates processing speed, visuomotor tracking, and sustained attention. Part B assesses set-shifting, divided attention, working memory, and cognitive flexibility [27]. Longer completion times reflect poorer performance.
- Rey–Osterrieth Complex Figure Test (RCFT): Measures visuoconstructional ability, planning and organizational strategies, and visual memory (immediate and delayed recall). Provides quantitative indices sensitive to executive function and nonverbal memory deficits [28].
- Brief Cognitive Screening Battery (BCSB): Evaluates naming, incidental learning, immediate and delayed recall, recognition memory, and visuoconstructional abilities. Clock drawing assesses executive planning and visuospatial processing and is scored using the Sunderland criteria [29, 30].
- Wechsler Abbreviated Scale of Intelligence (WASI): Provides estimates of general intellectual functioning (FSIQ), verbal reasoning, and nonverbal/problem-solving abilities through the Vocabulary, Block Design, Similarities, and Matrix Reasoning subtests [31].

Clinical and Behavioral Scales:

- Barkley Deficits in Executive Functioning Scale – Short Form (BDEFS-SF): Self-report measure assessing executive functions in daily life, including time management, organization/problem-solving, self-restraint, self-motivation, and emotion regulation. Age-adjusted scores between the 20th–80th percentiles are considered normal [32].
- Hospital Anxiety and Depression Scale (HADS): Assesses severity of anxiety (HADS-A) and depression (HADS-D) symptoms. Interpretation: 0–7 minimal; 8–11 mild–moderate; 12–21 marked symptom severity. Cutoffs: ≥8 for anxiety, ≥ 9 for depression [33]. Not diagnostic—reflects symptom intensity only.

### Intervention: Cognitive Training

During a home visit, the patient and caregivers were introduced to the NeuroNation^®^ platform (Synaptikon GmbH, Berlin, Germany), which was selected as the cognitive training tool for this study. Licenses for platform access were provided free of charge by Synaptikon GmbH. Initially, patients were instructed to install the application on mobile phones, computers, or tablets. The app is freely available for Android^®^ (OHA, USA) and iOS^®^ (Apple Inc., Cupertino, USA), and participants were guided to enter an access code to unlock the complete set of games [34].

NeuroNation^®^ is a platform dedicated to cognitive training and mental development, grounded in scientific research on neuroplasticity. Developed in partnership with the Free University of Berlin (Freie Universität Berlin – FU Berlin, Germany), the platform offers a variety of games designed to stimulate different brain functions, including memory, attention, executive function, and motor coordination. Specifically, the platform includes:

- 8 attention exercises (e.g., Clockwise, Color Craze, Form Fever, Parallel Perfection, Parita, Quantum Leap, Quick Switch, Speed of Light);
- 14 memory exercises (e.g., Chain Reaction, Flash Memo, Focus Master, Mathrobatics, Memo Mart, Memo Pair, Memobox, Memoflow, Path Finder, Path Finder Reverse, Rapid Recall (Beta), Reflector, Restorer, Shuffler, Turnabout, Word Memobox);
- 13 executive function exercises (e.g., Form Fusion, Formula, Logiladder, Rotator, Solitaria, Trail Tracker, Alphabet Soup, Flash Glance, Math Blitz, Symbolism, Verbal Voyager, Word Acrobat, Wordsmith).

Participants had unrestricted access to the full suite of games on the NeuroNation^®^ platform. Upon initial login, the system conducted a baseline cognitive assessment and generated a personalized training plan based on each participant’s cognitive profile. Participants were instructed to complete training three times a week for six months, with adaptive difficulty levels adjusted according to individual performance. Adherence was monitored through weekly follow-ups via messages, phone calls, and home visits as needed. At the end of the training period, Synaptikon GmbH (NeuroNation^®^) provided individual performance reports.

### Statistical Analysis

Results were presented as means, medians, standard deviations, and confidence intervals (for quantitative variables) and as frequencies and percentages (for categorical variables), based on the sample profile. The normality of continuous variables was assessed using the Shapiro-Wilk test. The 2-tailed Student’s t-test was employed to compare groups on parametric quantitative variables, and a paired 2-tailed Student’s t-test was used to compare parametric quantitative variables between baseline and post-intervention assessments. The appropriate chi-square or Fisher’s exact test was used for comparisons involving categorical variables. The Benjamini-Hochberg method was used to estimate the False Discovery Rate (FDR) for multiple-comparison correction. Spearman’s correlation coefficient was calculated to assess associations between variables. Correlation strength was interpreted as follows: very strong (> 0.9), strong (0.7–0.89), moderate (0.4–0.69), weak (0.2–0.39), and very weak (0.0–0.19). A *p*-value of < 0.05 was considered statistically significant. All statistical analyses were performed using Microsoft Excel (version 2010) and the open-source statistical software R (version 3.4.1).

## Results

The individuals with SCA2 belonged to five distinct families. Their clinical and epidemiological characteristics, as well as family pedigrees, are presented in Supplementary Material 1.

### Patients vs. Controls

There were no significant differences between the SCA2 and control groups in age (*p* = 0.89) or educational level (*p* = 0.94), as shown in Table 1. There was a worsening of motor severity as assessed by the SARA scale, indicating significant disease progression over the study period (*p* < 0.0001; Table 1).

**Table 1:** Demographic, Clinical, and neuropsychological characteristics of the sample

Variables	Patients (*n* = 12)	Controls (*n* = 12)	*p*-value*	*p*-value with FDR^#^
Sex (Male)	6 (50%)	6 (50%)	1**	**1**
Age (years)	46 ± 11.51	47 ± 11.68	0.847	**0.949**
Education (years)	8 ± 3.69	8 ± 3.85	0.789	**0.94**
Age at disease onset (years)	38.16 ± 10.14	-		
Disease duration (years)	9 ± 6.49	-		
Motor severity (SARA)				
Study onset	12 ± 5.62	-	< 0.0001***	**0.002**
Study endpoint	16 ± 7.29	-		
HAD Scale				
Anxiety symptoms	7.42 ± 3.78	7.92 ± 6.19	0.813	**0.94**
Depressive symptoms	6.42 ± 2.75	6.33 ± 5.60	0.963	**1**
Cognitive Tests				
MoCA-B	21.92 ± 4.83	29.5 ± 0.52	**< 0.001**	**0.002**
Clock Drawing	6.17 ± 2.48	6.08 ± 2.27	**< 0.001**	**0.002**
Verbal Fluency				
Semantic (animals)	11 ± 2.86	16.58 ± 2.64	**< 0.001**	**0.002**
Phonemic (F A S)	20.33 ± 7.32	54.17 ± 13.02	**< 0.001**	**0.002**
Trail Making Test				
Part A	132.25 ± 98.82	32.08 ± 10.76	**0.002**	**0.003**
Part B	320.17 ± 255.31	68.65 ± 27.98	**0.002**	**0.003**
Rey-Osterrieth Complex Figure Test				
Copy (Score)	23.67 ± 7.28	35.83 ± 0.39	**< 0.001**	**0.002**
Copy Time (min)	6.08 ± 4.66	3.5 ± 2.20	0.096	0.131
STM (3 min)	8 ± 4.71	26.92 ± 7.10	**< 0.001**	**0.002**
STM Recall Time	2.42 ± 1.88	2.67 ± 1.44	0.71	0.875
LTM (25 min)	7.71 ± 4.77	25.92 ± 6.10	**< 0.001**	**0.002**
BDEFS-SF				
Time Management	7 ± 1.65	10.25 ± 4.18	**0.02**	**0.03**
Self-Control	7017 ± 2.72	5.42 ± 1.83	0.07	0.103
Self-Regulation	7.67 ± 2.61	8.5 ± 2.65	0.44	0.561
Organization	9.83 ± 2.12	7.08 ± 2.75	**0.01**	**0.016**
Motivation	6.08 ± 1.83	6 ± 3.57	0.94	1
Symptoms	5.50 ± 2.15	4.33 ± 4.52	0.42	0.554
Total Score	37.75 ± 3.86	33.25 ± 7.81	0.08	0.113
Brief Cognitive Battery				
Naming	10 ± 0	10 ± 0	1	1
Incidental Memory	4.25 ± 1.60	5 ± 1.71	**0.006**	**0.01**
Immediate Memory 1	5.58 ± 1.62	6.58 ± 1.93	**< 0.001**	**0.002**
Immediate Memory 2	6 ± 1.60	7.17 ± 1.80	**< 0.001**	**0.002**
Delayed Memory	5.50 ± 1.78	6.83 ± 1.99	**< 0.001**	**0.002**
Recognition Memory	7.42 ± 2.1	8.42 ± 1.7	**< 0.001**	**0.002**
WASI Vocabulary Similarities Block Design Matrix Reasoning Verbal IQ Performance IQ Full-Scale IQ	32.25 ± 6.90 36.75 ± 6.73 34.08 ± 7.96 32 ± 6.31 74 ± 10.47 71.33 ± 10.84 69.08 ± 8.88	57.75 ± 5.43 57.25 ± 3.82 57.5 ± 7.83 59.67 ± 6.12 112 ± 7.51 114.08 ± 11.08 115.5 ± 7.13	**< 0.001** **< 0.001** **< 0.001** **< 0.001** **< 0.001** **< 0.001** **< 0.001**	**0.002** **0.002** **0.002** **0.002** **0.002** **0.002** **0.002**

*n = number of participants*, %=frequency, SARA = Scale for the Assessment and Rating of Ataxia, STM = Short-Term Memory; LTM = Long-Term Memory; BDEFS-SF = Barkley Deficits in Executive Functioning Scale—Short Form; MoCA-B = Montreal Cognitive Assessment—Basic; WASI = Wechsler Abbreviated Scale of Intelligence

* Student’s 2-tailed t-test for independent samples. Exceptions: **Fisher’s Exact Test; *** Paired 2-tailed Student’s t-test

^**#**^FDR (False Discovery Rate) calculated by the Benjamini-Hochberg method

The baseline neuropsychological assessment showed that the SCA2 group performed significantly worse than the control group across nearly all measures. On the MoCA-B, SCA2 patients scored an average of 7.58 points lower than the control group (*p* < 0.01). While the BDEFS-SF, a gold-standard measure of executive function, did not show statistically significant group differences, this finding was not consistent across the other executive assessments. Complementary measures—including the executive function subfunctions of the MoCA-B, the clock drawing test, and TMT A and B—demonstrated clear and significant executive dysfunction in SCA2 patients. Immediate and delayed recall tasks also showed marked impairments, with results from additional memory assessments corroborating deficits across multiple functions, particularly as measured by the BCSB (Table 1).

### Before and after Training

All participants adhered to the minimum required training frequency, as confirmed by platform usage logs. After six months, they underwent repeat neuropsychological assessments.

For measures of executive function and visuospatial processing, completion times on both the TMT Part A and Part B increased significantly (*p* < 0.01 for both), indicating a worsening in processing speed and cognitive flexibility (Table 2).

**Table 2:** Comparison of experimental group participants before and after cognitive training

Cognitive Test	Patients undergoing cognitive training Before (*n* = 12)	Patients undergoing cognitive training After (*n* = 12)	*p*-value*	*p*-value with FDR**
MoCA-B	21.92 ± 4.83	24.17 ± 3.61	0.09	0.2
Clock Drawing	6.17 ± 2.48	6.08 ± 2.27	0.82	0.937
Verbal Fluency Semantic (animals) Phonemic (F A S)	11 ± 2.86 20.33 ± 7.32	10.92 ± 4.72 18.42 ± 5.07	0.95 0.36	0.98 0.548
Trail Making Test Part A Part B	132.25 ± 98.82 320.17 ± 255.31	178.50 ± 119.25 388.67 ± 244.65	**0.002** **0.002**	**0.009** **0.009**
Rey-Osterrieth Complex Figure Test Copy (Score) Copy Time (min) STM (3 min) STM Recall Time LTM (25 min)	23.67 ± 7.28 6.08 ± 4.66 8 ± 4.71 2.42 ± 1.88 7.71 ± 4.77 1.92 ± 1	25.79 ± 6.35 8.42 ± 5.12 9.29 ± 4.49 3.42 ± 1.93 8.13 ± 4.85 2.50 ± 1.62	0.251 **0.001** 0.151 **0.02** 0.59 0.171	0.42 **0.009** 0.3 0.064 0.82 0.3
BDEFS-SF Time Management Self-Control Self-Regulation Organization Motivation Symptoms Total Score	7 ± 1.65 7.17 ± 2.72 7.67 ± 2.61 9.83 ± 2.12 6.08 ± 1.83 5.50 ± 2.15 37.75 ± 3.86	6.67 ± 1.78 12.50 ± 2.07 5.75 ± 2.30 6.58 ± 2.23 7.08 ± 1.98 5.58 ± 1.88 38.58 ± 5.05	0.65 **< 0.001** 0.13 **0.002** 0.25 0.92 0.68	0.866 **0.003** 0.27 **0.009** 0.421 0.98 0.87
Brief Cognitive Battery Naming Incidental Memory Immediate Memory 1 Immediate Memory 2 Delayed Memory Recognition Memory	10 ± 0 4.25 ± 1.60 5.58 ± 1.62 6 ± 1.60 5.50 ± 1.78 7.42 ± 2.15	10 ± 0 5 ± 1.71 6.58 ± 1.93 7.17 ± 1.80 6.83 ± 1.99 8.42 ± 1.78	1 **0.03** **0.006** **0.002** **0.001** **0.015**	1 0.08 **0.024** **0.009** **0.009** 0.053
WASI Vocabulary Similarities Block Design Matrix Reasoning Verbal IQ Performance IQ Full-Scale IQ	32.25 ± 6.90 36.75 ± 6.73 34.08 ± 7.96 32 ± 6.31 74 ± 10.47 71.33 ± 10.84 69.08 ± 8.88	31 ± 3.81 36.50 ± 4.91 34.58 ± 4.87 34.67 ± 5.84 67.67 ± 7.92 69.25 ± 9.17 68.45 ± 7.36	0.57 0.85 0.74 **0.04** **0.03** 0.32 0.76	0.82 0.937 0.9 0.098 0.08 0.512 0.9

*n = number of participants*, STM = Short-Term Memory; LTM = Long-Term Memory; BDEFS-SF = Barkley Deficits in Executive Functioning Scale—Short Form; MoCA-B = Montreal Cognitive Assessment—Basic; WASI = Wechsler Abbreviated Scale of Intelligence

* Paired Student’s 2-tailed t-test

**FDR (False Discovery Rate) calculated by the Benjamini-Hochberg method

On the BDEFS-SF, significant improvement was observed in the Organization dimension (*p* < 0.01), while the Self-Control dimension showed a significant decline (*p* < 0.01). The total BDEFS-SF score remained unchanged (*p* = 0.866). In contrast, significant improvements in episodic memory were observed on the BCSB, with gains in immediate recall trials 1 (*p* < 0.05) and 2 (*p* < 0.01), as well as in delayed recall (*p* < 0.01) (Table 2). Memory improvements were not correlated with demographic or clinical variables, including education level, SARA score, age, or disease duration. The only exception was a moderate positive correlation between improvement in delayed memory and greater motor impairment, as reflected by the SARA score (*r* = 0.663, *p* = 0.018).

The NeuroNation^®^ platform automatically adjusted task difficulty according to individual performance. However, analysis of the highest difficulty levels revealed no strong correlations with neuropsychological test outcomes. A few moderate associations were identified, primarily in the function of verbal fluency and self-regulation. The strongest correlation was observed with semantic verbal fluency (animals) (*r* = 0.407, *p* = 0.188), though it did not reach statistical significance. On the BDEFS-SF, moderate correlations were found in the Self-Regulation dimension (*r* = −0.515, *p* = 0.086) and the Symptoms dimension (*r* = −0.454, *p* = 0.137). All other cognitive functions demonstrated weak or very weak correlations with performance on the platform.

## Discussion

The results of this study demonstrated statistically significant differences between the experimental and control groups across multiple cognitive functions, with marked impairments in verbal memory, visuospatial memory, and executive function. These findings are consistent with previous literature describing progressive neurocognitive decline in patients with SCA2 [10, 35]. Following the cognitive training intervention, participants demonstrated significant improvements in memory, the functions most severely affected prior to the intervention [10, 22, 23]. However, no meaningful improvement in executive function was observed, suggesting that memory may be more responsive to cognitive training in this population.

Although a significant impairment in executive functions was expected, only select measures within this function demonstrated change. Notably, scores on the more behaviorally oriented dimensions of the BDEFS-SF were comparable between groups, suggesting that executive dysfunction in SCA2 may extend beyond cognitive task performance to affect behavioral regulation and environmental adaptability. In contrast, semantic and phonemic verbal fluency tests revealed marked impairments in the SCA2 group [4, 5]. These findings are consistent with those reported by Clausi et al. [4], Olivito et al. [36], and Rodríguez-Labrada et al. [5], who identified phonemic fluency as a sensitive marker of cerebellar-related executive dysfunction, reflecting deficits in lexical retrieval, verbal planning, inhibitory control, and response monitoring. These impairments are thought to arise from cortico-cerebellar disruptions affecting higher-order cognitive processes, including language, in SCA2 patients [4].

Performance on the TMT confirmed the presence of executive dysfunction, with significant slowing in both Parts A and B. The marked impairment in Part B, which primarily evaluates cognitive flexibility and set-shifting, supports the presence of executive deficits [27]. Although motor incoordination can contribute to slower performance, specific measures were taken to minimize its confounding effects on cognitive testing. Notably, in the Rey-Osterrieth Complex Figure Test, copy time was recorded but intentionally excluded from scoring, in accordance with standardized procedures, thereby reducing the impact of motor speed on task performance. This approach strengthens the interpretation that the slowing observed on the TMT reflects genuine cognitive inefficiency rather than motor deterioration alone.

BCSB results further confirmed widespread memory impairment, supporting previous studies that emphasized cognitive deficits in SCA2 beyond executive dysfunction [37, 38]. Verbal memory impairment was particularly prominent, affecting all phases from incidental encoding to delayed recognition, reinforcing the hypothesis of impaired encoding and consolidation of verbal information. In SCA2, cortico-cerebellar dysfunction—particularly involving posterior cerebellar connections with parietal and temporal cortices—may disrupt the integration of sensory, spatial, and mnemonic information [37, 38].

Memory was the cognitive function that showed the most robust improvement following the intervention, particularly in immediate and delayed recall. These results align with previous findings suggesting that digital cognitive training yields greater effects on memory and reasoning than on executive functions [19]. Memory processes that rely less on executive regulation and more on automated encoding and retrieval benefit most from repetitive, structured training. Significant improvements in the BCSB memory subtests support enhanced encoding, consolidation, and retrieval, with naming remaining stable. RCFT results reinforced persistent difficulties in visuoconstructive organization but demonstrated gains in recall, consistent with prior studies linking cerebellar dysfunction to impairments in memory sequencing and retrieval strategies [39]. The differential response between memory and executive functions may reflect the more targeted nature of memory-oriented tasks compared to executive tasks, which depend on broader and more distributed neural networks. Similar training-related memory gains have been reported in PD cohorts using the NeuroNation^®^ platform [21–23].

No improvements were observed in verbal fluency or other executive function measures, consistent with prior studies showing that executive functions—specifically, verbal initiation and inhibitory control—are less responsive to repetitive, low-context training because they rely on fronto-cerebellar circuits [4, 5]. While the BDEFS-SF showed improvement in the organization dimension, a paradoxical decline in self-control was noted, possibly reflecting increased metacognitive awareness of executive deficits or cognitive fatigue related to training demands [4, 17, 37]. WASI subtests remained largely stable, with slight gains in Matrix Reasoning, suggesting preserved plasticity in fluid intelligence despite persistent executive dysfunction [19].

This study has several limitations. The relatively small sample size, combined with heterogeneity in disease duration, severity, and CAG repeat length, limits statistical power and generalizability. Although patients with overt behavioral disturbances were excluded, the assessment tools employed focused exclusively on depressive and anxiety symptoms. As a result, milder psychotic features, sleep disturbances, or other psychiatric conditions may have gone undetected, and these factors could potentially influence cognitive performance. The six-month follow-up period may also have been too brief to capture subtle or long-term cognitive effects. Although the NeuroNation^®^ adaptive algorithm was designed to balance cognitive functions, individual engagement patterns may have led to an overrepresentation of memory-oriented tasks, potentially understimulating executive and visuospatial processes. Nevertheless, the absence of strong correlations between training metrics and cognitive outcomes suggests that factors such as disease stage, cognitive reserve, and motivation may exert a greater influence on responsiveness than nominal task difficulty alone. Another limitation is the absence of multimodal biomarkers, which restricts the mechanistic interpretation of the findings. Future studies integrating neuroimaging and fluid biomarkers—such as cerebellar and cerebral volumetry (particularly of Crus I/II), diffusion tractography of cerebello-thalamo-cortical pathways, resting-state functional connectivity, and neurofilament or synaptic markers—could help disentangle motor from cognitive contributions and identify responders to intervention [14, 35, 37].

In conclusion, this study suggests that patients with SCA2 exhibit cognitive deficits across neurocognitive functions, with memory dysfunction emerging as a particularly prominent—and potentially modifiable—feature through digital cognitive training. These findings highlight the potential utility of targeted interventions in mitigating specific cognitive deficits. However, further longitudinal studies with larger samples and extended follow-up are needed to confirm these results and to more comprehensively assess the impact of cognitive training on executive function, attention, language, visuospatial processing, and the long-term trajectory of cognitive decline in SCA2.

## Supplementary Information

Below is the link to the electronic supplementary material.

Supplementary Material 1 (DOCX 21.3 KB)

## Data Availability

No datasets were generated or analysed during the current study.
