# Supplementary material for: Targeting Cognitive Dysfunction in Spinocerebellar Ataxia Type 2 Through Digital Cognitive Training
Source: Cerebellum. 2026 Mar 2;25(2):28. doi: 10.1007/s12311-025-01956-2 (PMC12953462; doi:10.1007/s12311-025-01956-2)
Supplement: Supplementary file 1 — Supplementary Material 1 (DOCX 21.3 KB) [file 12311_2025_1956_MOESM1_ESM.docx]

Supplementary Material 3

| Test | Mean ± SD (SCA2) | Mean ± SD (Controls) | p | t | F | Cohen’s d |
| --- | --- | --- | --- | --- | --- | --- |
| Age (years) | 46 ± 11.51 | 47 ± 11.68 | 0.847 | -0.194 | 0.04 | 0.086 |
| Education (years) | 8 ± 3.69 | 8 ± 3.85 | 0.789 | 0.27 | 0.07 | 0.052 |
|  |  |  |  |  |  |  |
| HADS – Anxiety | 7.42 ± 3.78 | 7.92 ± 6.19 | 0.813 | -0.24 | 0.06 | 0.10 |
| HADS – Depression | 6.42 ± 2.75 | 6.33 ± 5.60 | 0.963 | 0.046 | 0.002 | 0.02 |
|  |  |  |  |  |  |  |
| MoCA-B | 21.92 ± 4.83 | 29.5 ± 0.52 | <0.001 | -5.88 | 34.6 | 1.86 |
| Clock Drawing | 6.17 ± 2.48 | 6.08 ± 2.27 | <0.001 | 0.11 | 0.01 | 0.038 |
|  |  |  |  |  |  |  |
| Semantic Fluency | 11 ± 2.86 | 16.58 ± 2.64 | <0.001 | -5.86 | 34.4 | 1.99 |
| Phonemic Fluency | 20.33 ± 7.32 | 54.17 ± 13.02 | <0.001 | -7.89 | 62.3 | 3.02 |
|  |  |  |  |  |  |  |
| TMT-A (s) | 132.25 ± 98.82 | 32.08 ± 10.76 | 0.002 | 3.44 | 11.8 | 1.47 |
| TMT-B (s) | 320.17 ± 255.31 | 68.65 ± 27.98 | 0.002 | 3.44 | 11.8 | 1.47 |
|  |  |  |  |  |  |  |
| ROCF – Copy Score | 23.67 ± 7.28 | 35.83 ± 0.39 | <0.001 | -6.36 | 40.5 | 2.03 |
| ROCF – Copy Time | 6.08 ± 4.66 | 3.5 ± 2.20 | 0.096 | 1.74 | 3.0 | 0.67 |
| STM (3 min) | 8 ± 4.71 | 26.92 ± 7.10 | <0.001 | -8.36 | 69.9 | 3.18 |
| STM – Recall Time | 2.42 ± 1.88 | 2.67 ± 1.44 | 0.71 | -0.38 | 0.14 | 0.15 |
| LTM (25 min) | 7.71 ± 4.77 | 25.92 ± 6.10 | <0.001 | -8.30 | 68.9 | 3.26 |
|  |  |  |  |  |  |  |
| BDEFS – Time Mgmt | 7 ± 1.65 | 10.25 ± 4.18 | 0.02 | -2.58 | 6.66 | 0.97 |
| BDEFS – Self Control | 7.17 ± 2.72 | 5.42 ± 1.83 | 0.07 | 1.92 | 3.68 | 0.74 |
| BDEFS – Self-Regulation | 7.67 ± 2.61 | 8.5 ± 2.65 | 0.44 | -0.78 | 0.61 | 0.31 |
| BDEFS – Organization | 9.83 ± 2.12 | 7.08 ± 2.75 | 0.01 | 3.05 | 9.30 | 1.11 |
| BDEFS – Motivation | 6.08 ± 1.83 | 6 ± 3.57 | 0.94 | 0.078 | 0.006 | 0.03 |
| BDEFS – Symptoms | 5.50 ± 2.15 | 4.33 ± 4.52 | 0.42 | 0.82 | 0.67 | 0.32 |
| BDEFS – Total | 37.75 ± 3.86 | 33.25 ± 7.81 | 0.08 | 1.87 | 3.49 | 0.72 |
|  |  |  |  |  |  |  |
| BCSB – Naming | 10 ± 0 | 10 ± 0 | 1 | – | – | – |
| BCSB – Incidental Mem | 4.25 ± 1.60 | 5 ± 1.71 | 0.006 | -2.99 | 8.94 | 1.04 |
| BCSB – Immediate 1 | 5.58 ± 1.62 | 6.58 ± 1.93 | <0.001 | -3.72 | 13.8 | 1.30 |
| BCSB – Immediate 2 | 6 ± 1.60 | 7.17 ± 1.80 | <0.001 | -3.91 | 15.3 | 1.34 |
| BCSB – Delayed | 5.50 ± 1.78 | 6.83 ± 1.99 | <0.001 | -3.36 | 11.3 | 1.15 |
| BCSB – Recognition | 7.42 ± 2.10 | 8.42 ± 1.70 | <0.001 | -3.10 | 9.61 | 1.06 |
|  |  |  |  |  |  |  |
| WASI – Vocabulary | 32.25 ± 6.90 | 57.75 ± 5.43 | <0.001 | -10.8 | 117 | 4.07 |
| WASI – Similarities | 36.75 ± 6.73 | 57.25 ± 3.82 | <0.001 | -11.1 | 123 | 4.14 |
| WASI – Block Design | 34.08 ± 7.96 | 57.5 ± 7.83 | <0.001 | -7.75 | 60.1 | 2.80 |
| WASI – Matrix | 32 ± 6.31 | 59.67 ± 6.12 | <0.001 | -12.0 | 144 | 4.42 |
| WASI – Verbal IQ | 74 ± 10.47 | 112 ± 7.51 | <0.001 | -10.3 | 107 | 3.82 |
| WASI – Perf IQ | 71.33 ± 10.84 | 114.08 ± 11.08 | <0.001 | -10.1 | 102 | 3.72 |
| WASI – Full-Scale IQ | 69.08 ± 8.88 | 115.5 ± 7.13 | <0.001 | -13.8 | 190 | 4.93 |
